# Murine Cytomegalovirus Disrupts Splenic Dendritic Cell Subsets *via* Type I Interferon-Dependent and -Independent Mechanisms

**DOI:** 10.3389/fimmu.2017.00251

**Published:** 2017-03-09

**Authors:** William T. Nash, Alyssa L. Gillespie, Michael G. Brown

**Affiliations:** ^1^Department of Microbiology, Immunology, and Cancer Biology, School of Medicine, University of Virginia, Charlottesville, VA, USA; ^2^Beirne B. Carter Center for Immunology Research, School of Medicine, University of Virginia, Charlottesville, VA, USA; ^3^Division of Nephrology, Department of Medicine, University of Virginia, Charlottesville, VA, USA

**Keywords:** MCMV, IFN-I, licensed NK cell, spleen DC subsets, DC loss, CD8^+^ DC, CD4^+^ DC, DN DC

## Abstract

Dendritic cells (DC) are well-known modulators of immunity. This heterogeneous population is composed of defined subsets that exhibit functional specialization and are critical in initiating responses to pathogens. As such, many infectious agents employ strategies to disrupt DC functioning in attempts to evade the immune system. In some instances, this manifests as an outright loss of these cells. Previous work has suggested that, in the absence of an efficient natural killer (NK) cell response, murine cytomegalovirus (MCMV) induces large amounts of interferon (IFN)-I. This heightened IFN-I response is thought to contribute to conventional DC (cDC) loss and delayed development of T cell immunity. However, the precise role of IFN-I in such cDC loss remains unclear. We investigated the effects of licensed NK cells and IFN-I signaling on splenic cDC subsets during MCMV infection and found that a licensed NK cell response partially protects cDC numbers, but does not prevent increases in serum IFN-I. This suggested that high residual IFN-I could contribute to cDC loss. Therefore, we used multiple strategies to modulate IFN-I signaling during MCMV infection including plasmacytoid DC depletion, IFN-I receptor (IFNAR) blockade, and genetic ablation of IFNAR expression. Interestingly, restriction of IFN-I signals did not substantially preserve either CD8^+^ or CD4^+^ DC total numbers, but resulted in significant retention and/or accumulation of the splenic CD8^−^ CD4^−^ [double negative (DN)] subset. However, the DN DC effect manifested in a DC-extrinsic manner since IFNAR-deficient cells were not preferentially retained over their IFNAR wild-type counterparts in a mixed-chimera setting. Our results show that IFN-I signaling is not responsible for overt cDC toxicity in the setting of acute MCMV infection and emphasize that additional mechanisms contribute to DC loss and require exploration.

## Introduction

Due to their key involvement in pathogen detection, innate responses, and adaptive immunity, dendritic cells (DC) are prime targets for suppression by viral infections (1, 2). Although it is well established that the type I interferon (IFN)-I family plays important roles in the initial control of many viral infections (3, 4), previous mouse studies have also suggested a causal role for IFN-I in splenic DC loss during infection or toll-like receptor stimulation (5–11). However, the exact contribution of IFN-I to DC loss is still unclear. It is thought that collapse of the DC compartment in such contexts can contribute to infection-induced immunosuppression at later time points. Previous work has shown that inefficient early control of murine cytomegalovirus (MCMV) leads to loss of DC numbers and can delay virus-specific CD8 T cell priming and memory precursor formation (5, 12, 13). If the process underlying DC loss is IFN dependent, a similar mechanism may help explain aspects of immunosuppression observed in human conditions marked by heightened inflammation, like HIV and sepsis (14–18).

Additional work has shown that natural killer (NK) cells can limit inflammation and protect DC—at least in the setting of MCMV infection (5, 8, 9). NK cell-dependent protection of DC in this setting is presumed to stem from their ability to limit viral spread and replication, thereby restricting the source material for plasmacytoid DC (pDC) stimulation and downstream IFN-I production (5, 10). Notably, many of the previous studies examining DC numbers in the context of MCMV control have been performed in mice with Ly49H^+^ NK cells. Ly49H specifically binds MCMV-encoded m157 protein on the surface of infected cells, enabling NK cells to rapidly eliminate virally infected targets (19–23). However, NK cell-mediated control also results from additional receptor–ligand interactions. For example, NK cells that express the Ly49G2 inhibitory receptor (G2) can enhance MCMV resistance in mice that express the MHC class I molecule D^k^ (12, 13, 24–26). D^k^ is a cognate ligand of Ly49G2 and prompts G2^+^ NK cells to undergo an education process. These educated, or licensed, NK cells acquire the capability to detect alterations in D^k^ expression and orchestrate a more potent anti-MCMV immune response (12, 13, 24–26). The exact mechanism underlying this enhanced resistance is still under investigation, but the licensed, G2^+^ NK cell population is critical to the effect. Previous observations have indicated that this manifestation of MCMV control does not preserve DC to the same extent as a Ly49H-dependent response, but the role of IFN-I in the sustained DC loss is still unknown (12).

It is also unclear to what extent IFN-I affects individual subpopulations of DC during *in vivo* MCMV infection. It is now known that the mouse spleen contains at least four distinct groups of DC, composed of pDC and three subsets of resident conventional DC (cDC). These distinct DC populations are distinguished by both their expression of surface molecules and their functional specializations. The pDC subtype is characterized by lower expression of CD11c and MHC II (compared to cDC), high expression of B220 and mouse pDC antigen (mPDCA), the capability to quickly produce large amounts of IFN-I, and a reduced ability to efficiently prime T cell responses (27, 28). The bulk cDC population is broadly classified as CD11c-hi/MHC II^+^, and in the spleen and other lymphoid tissues, resident cDC subsets can be generally defined by their expression of CD8, CD11b, and CD4. The CD8^+^ DC subset (CD11b^−^ CD8^+^ CD4^−^) is specialized for the uptake of dead cells and cross-presentation of extracellular material to CD8^+^ T cells. These cells are critical for efficient CD8^+^ T cell priming in response to certain viral infections and immunogenic tumors (29). CD4^+^ DC (CD11b^+^ CD8^−^ CD4^+^) are more efficient *in vivo* stimulators of CD4^+^ T cell responses, and their intestinal equivalents are required for a robust immune response to attaching and effacing bacteria (30, 31). Little is known about the functional specialization of double-negative (DN) DC (CD11b^+^ CD8^−^ CD4^−^), but recent data indicate that these cells are superior cytokine producers compared to their CD4^+^ counterparts (30). Much progress has been made in identifying the transcription factors and developmental requirements for individual DC subsets (28), but our understanding of their regulation during infection and inflammation is still far from complete. Since IFN-I is known to cause pDC loss (32) and there is evidence indicating that IFN-I plays a role in cDC attrition (5, 6, 11), a deeper investigation into the requirement for this cytokine family in the context of cDC loss is warranted.

All members of the IFN-I cytokine family signal through a common IFN-I receptor (IFNAR). Therefore, the contribution of IFN-I signaling to a specific phenotype can be efficiently investigated by disrupting the association between IFN-I and IFNAR (33–38). As a natural mouse pathogen known to induce high levels of IFN-I and to drive splenic DC loss (5, 8), MCMV infection represents an ideal setting to assess the impacts of IFN-I signaling on DC subset loss. Here, we investigated the effects of MCMV, NK cell control, and IFN-I signaling on cDC numbers during acute MCMV infection. We found that, although cDC were partially protected in mice with G2^+^ NK-dependent resistance, IFN-I levels increased substantially in all mice during infection. Further investigation into the precise role of IFN-I revealed that CD8^+^ and CD4^+^ DC subset loss can occur independently of IFN-I stimulation, but DN DC numbers benefit from the removal of IFN-I signals.

## Materials and Methods

### Mice

C57L-derived MHC I D^k^-disparate congenic mouse strains R7 and R2 (referred to as D^k^ and non-D^k^, respectively) were previously generated and described (39). C57Bl/6 (B6)-derived IFNAR-KO mice (B6.129S2-*Ifnar1^tm1Agt^*/Mmjax) were obtained from the Mutant Mouse Resource & Research Centers. CD45.1 (45.1) mice (B6.SJL-*Ptprc^a^Pepc^b^*/BoyJ) were purchased from The Jackson Laboratory. Congenic B6 mice harboring the natural killer gene complex (NKC) haplotype from C57L mice (B6.NKC*^c57l^*; referred to here as NKC*^l^*) were generated and described previously (13). NKC*^l^* mice lack the *Ly49h* gene and, consequently, Ly49H^+^ NK cells (13). NKC*^l^* and IFNAR-KO mice were crossed to generate B6.Cg-*Nkc^c57l^*-129S2-*Ifnar1^tm1Agt^* (aka B6.NKC*^l^* IFNAR-KO). IFNAR wild-type (IFNAR-WT) and IFNAR-heterozygous (IFNAR-het) littermates from the crosses were included as comparators in experiments. This study was carried out in accordance with the recommendations of the Animal Welfare Act and the recommendations in the Guide for the Care and Use of Laboratory Animals of the National Institutes of Health. The protocol was approved by the University of Virginia Animal Care and Use Committee.

### MCMV Infection and Immune Cell Depletion

Mice were i.p. injected with salivary gland passaged MCMV (Smith strain ATCC VR1399; 5 × 10^4^ PFU/mouse, or as indicated). For depletion of pDC, mice were i.p. injected twice with 250 μg monoclonal antibody (mAb) 927 (kindly provided by Marco Colonna) (40) 48 and 24 h prior to infection. For depletion of G2^+^ NK cells, mice were i.p. injected with 200 μg mAb 4D11 (hybridoma kindly provided by Wayne Yokoyama) 48 h prior to infection. For blocking IFNAR, mice were i.p. injected with 1 mg mAb MAR1-5A3 (Leinco Technologies, Inc.) before infection and 500 μg every 24 h thereafter. For blocking Ly49H receptors, mice were i.p. injected with 200 μg mAb 3D10 (hybridoma also provided by Wayne Yokoyama) 24 h prior to infection.

### Splenic DC Harvest and Flow Cytometry

Disrupted spleen tissues were digested with collagenase D (Roche) and processed with Falcon cell strainers essentially as described (41). Splenocytes were Fc blocked with mAb 2.4G2 and stained with mAb purchased from eBiosciences, BioLegend, BD Pharmingen, and Miltenyi including 2G9 (MHC II), 53-6.7 (CD8), MAR1-5A3 (IFNAR1), 145-2C11 (CD3), 6D5 (CD19), P84 (SIRPα), N418 (CD11c), HL3 (CD11c), 129c1 (mPDCA), M1/70 (CD11b), GK1.5 (CD4), RA3-6B2 (B220), 53-2.1 (Thy1.2), A20 (CD45.1), 104 (CD45.2), and 30-F11 (pan-CD45). LIVE/DEAD^®^ Viability Dye (Invitrogen) was used for dead cell exclusion. Samples were run on a BD Canto II with Diva acquisition software and analyzed using FlowJo v10. Detailed gating strategies are illustrated in supplemental material (Figures S1 and S2 in Supplementary Material). Cell numbers were quantified *via* a series of calculations. Whole spleens and spleen fractions processed for flow cytometry were weighed to determine their mass. The number of splenocytes per flow sample was determined using a hemocytometer, and this was converted to splenocytes/g tissue. FlowJo data were then used to determine the number of DC/total splenocytes. This allowed us to calculate the number of DC/g tissue and then adjust for the total mass of the whole spleen.

A population of CD11c^+^ MHC II^+^ cells from infected mice was observed that displayed significantly increased CD11b expression and higher side scatter values. Thus, these cells were regarded as inflammatory monocytes/inflammatory DC and were excluded from analyses, as indicated in Figures S1 and S2 in Supplementary Material.

### IFNα ELISA

Blood was collected *via* tail bleed or postmortem cardiac puncture. Serum was isolated by allowing blood to clot followed by centrifugation. IFNα quantitation was performed with VeriKine Mouse IFN Alpha ELISA kits (PBL) according to the manufacturer’s instructions.

### Bone Marrow (BM) Chimeric Mice

Bone marrow transplantations were performed essentially as described (25). Briefly, B6 mice were irradiated twice with 5.5 Gy (11 Gy total) over a span of 3 h. Twenty-four hour later, i.v. injection of ~4.5 × 10^6^ total donor BM cells was performed, either IFNAR-KO (45.2), -WT (45.1), or a 1:1 mix. Recipient mice were maintained on sulfate drinking water for 3 weeks following irradiation. Peripheral blood analysis was performed at 4 weeks to assess BM chimerism. After 8 weeks, recipients were 3D10 treated to block the Ly49H receptor and i.p. infected 24 h later. Splenocytes were analyzed 3 days postinfection (d.p.i.). Due to limited flow cytometer channels in the BM chimera experiments, we defined CD8^+^ DC by low SIRPα and CD4 expression (28) to evaluate cDC subsets side by side. However, results were further confirmed by a separate analysis for CD8^+^ DC specifically (Figure S3 in Supplementary Material).

### Quantification of MCMV Genomes

Virus levels were measured by determining the ratio of viral genomes to β-actin in DNA isolated from tissue samples. DNA was isolated using the Gentra Puregene tissue kit according to the manufacturer’s instructions. Isolated DNA was analyzed by quantitative real-time PCR as described previously (42).

### Statistical Analysis

Since our samples sizes are not large enough to verify normality of the data, significance was determined with the non-parametric Mann–Whitney (two groups) or Kruskal–Wallis (more than two groups) tests using GraphPad Prism and XLSTAT software. If the Kruskal–Wallis test rejected the null hypothesis, a Conover–Iman *post hoc* test was run for multiple comparisons using XLSTAT. Significance is represented as follows: *p < 0.05, **p < 0.01, ***p < 0.001, and ****p < 0.0001. Error bars denote SD. Dots on graphs represent individual animals.

## Results

### Licensed NK Cell Protection of cDC during Acute MCMV Infection

**Figure 1:**
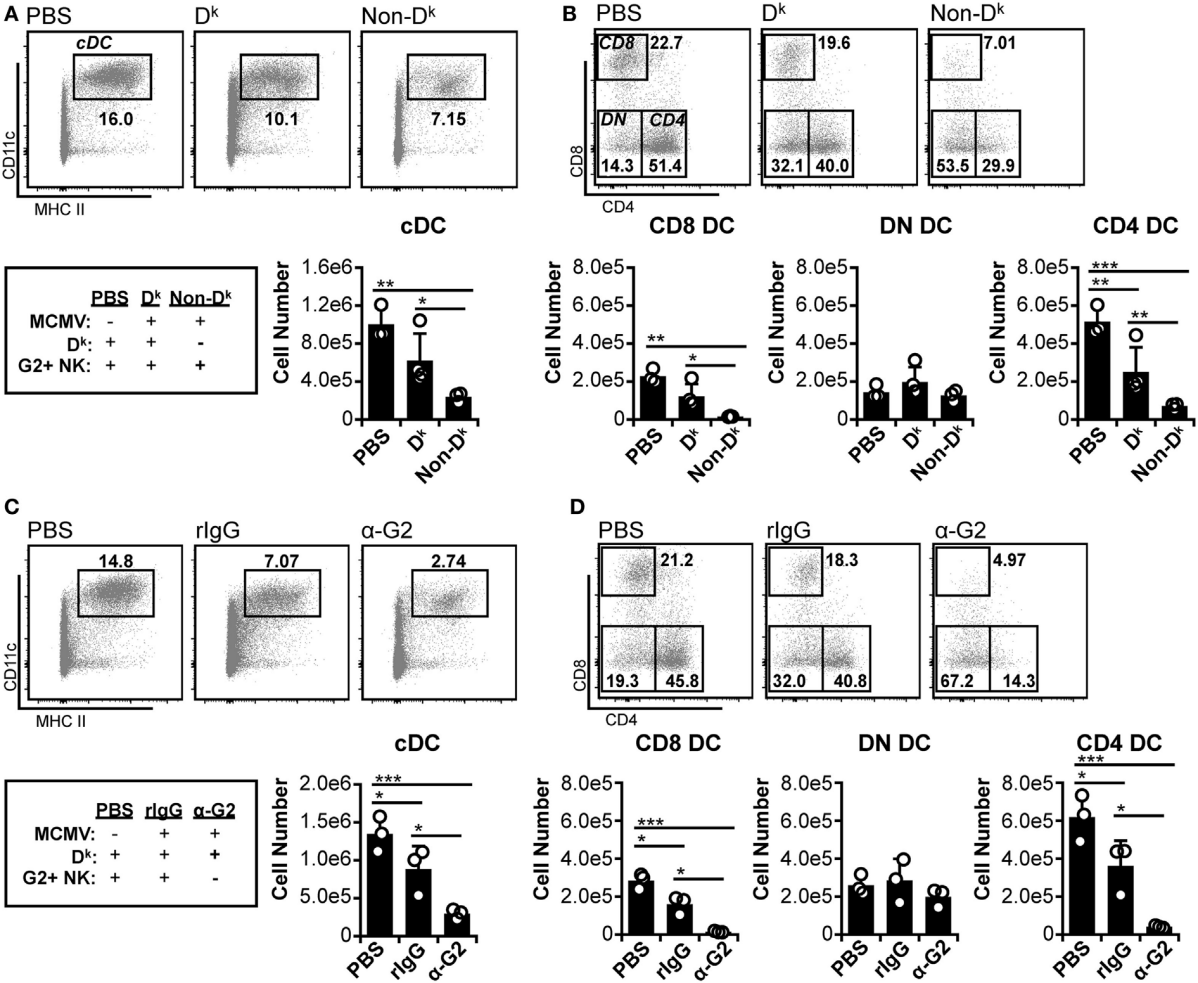
**Licensed natural killer (NK) cells partially protect conventional dendritic cell (cDC) during acute murine cytomegalovirus (MCMV) infection**. **(A,B)** D^k^ and non-D^k^ mice were mock (PBS) or MCMV infected for 3 days. Splenocytes were stained with fluorescent mAbs (mAb) and assessed for cDCs by flow cytometry. **(A)** Concatenated flow plots showing gated dendritic cells (DC) populations. DC numbers are quantified in the graph. **(B)** As in **(A)** except cDC subsets are analyzed. **(C,D)** D^k^ mice were treated with rIgG or mAb 4D11 to deplete Ly49G2^+^ NK cells (α-G2) 48 h prior to MCMV infection. Mice were mock (PBS) or MCMV infected for 3 days, and splenocytes were assessed for cDC. **(C)** Concatenated flow plots showing gated DC populations. DC numbers are quantified in the graph. **(D)** As in **(C)** except cDC subsets are analyzed. Numbers on flow plots denote frequency of parent population. Graphs show results for individual mice (dots) and group means (bars). Results are representative of at least two experiments.

Previous work has shown that Ly49H^+^ NK cells can preserve splenic cDC numbers during MCMV infection (5, 8, 9). However, the role of self-MHC I licensed NK cells in cDC protection has been minimally explored. We therefore examined the effect of D^k^-licensed G2^+^ NK cells on cDC populations during MCMV infection. Similar to prior work (12), we observed that total splenic cDC numbers declined by 3 d.p.i. in all mice (Figure 1A). The majority of loss occurred in the CD8^+^ and CD4^+^ DC subsets, whereas DN DC were largely unaffected (Figure 1B). However, overall cDC loss was less prominent in D^k^ mice with licensed G2^+^ NK cells, suggesting that the virus control mediated by these cells can enhance cDC retention. Both CD8^+^ and CD4^+^ DC numbers were higher in D^k^ mice compared to non-D^k^, but DN DC numbers were equivalent across all mice (Figure 1B). These results suggest that distinct cDC subsets are differentially sensitive to MCMV infection, and, as a result, licensed G2^+^ NK cell control differentially enhances subset numbers.

We further examined the importance of licensed NK cell-mediated control by specifically depleting G2^+^ NK cells prior to MCMV infection. Without Ly49G2^+^ NK cells, cDC loss was exacerbated in D^k^ mice compared to isotype-treated control mice; this was similar to the results obtained for non-D^k^ versus D^k^ mice (Figure 1C compared to Figure 1A). Individual cDC subset loss also mirrored the patterns from D^k^ and non-D^k^ mice. Reductions in cDC were observed in all infected mice, with CD8^+^ and CD4^+^ DCs constituting the entirety of cDC loss, while DN DC numbers remained unchanged (Figure 1D compared to Figure 1B). Loss of CD8^+^ and CD4^+^ DC was aggravated by the absence of G2^+^ NK cells, further demonstrating that an effective licensed NK cell response to MCMV can promote cDC retention. We considered the possibility that Ly49G2 depletion itself could have resulted in cDC loss, but no differences in DC numbers were detected following G2 depletion in the absence of infection (Figure S4A in Supplementary Material). We are also confident that protection is a Ly49G2-specific effect in these mice since all non-G2 NK cell subsets were preserved in G2-depleted mice (Figure S4B in Supplementary Material). Thus, CD8^+^ and CD4^+^ DCs are specifically sensitive to MCMV-induced loss, which is amplified in mice without licensed NK cell-mediated virus control.

### pDC Depletion Reduces Systemic IFN-I and Increases DN DC Numbers, but Does Not Restore CD8^+^ or CD4^+^ DC

**Figure 2:**
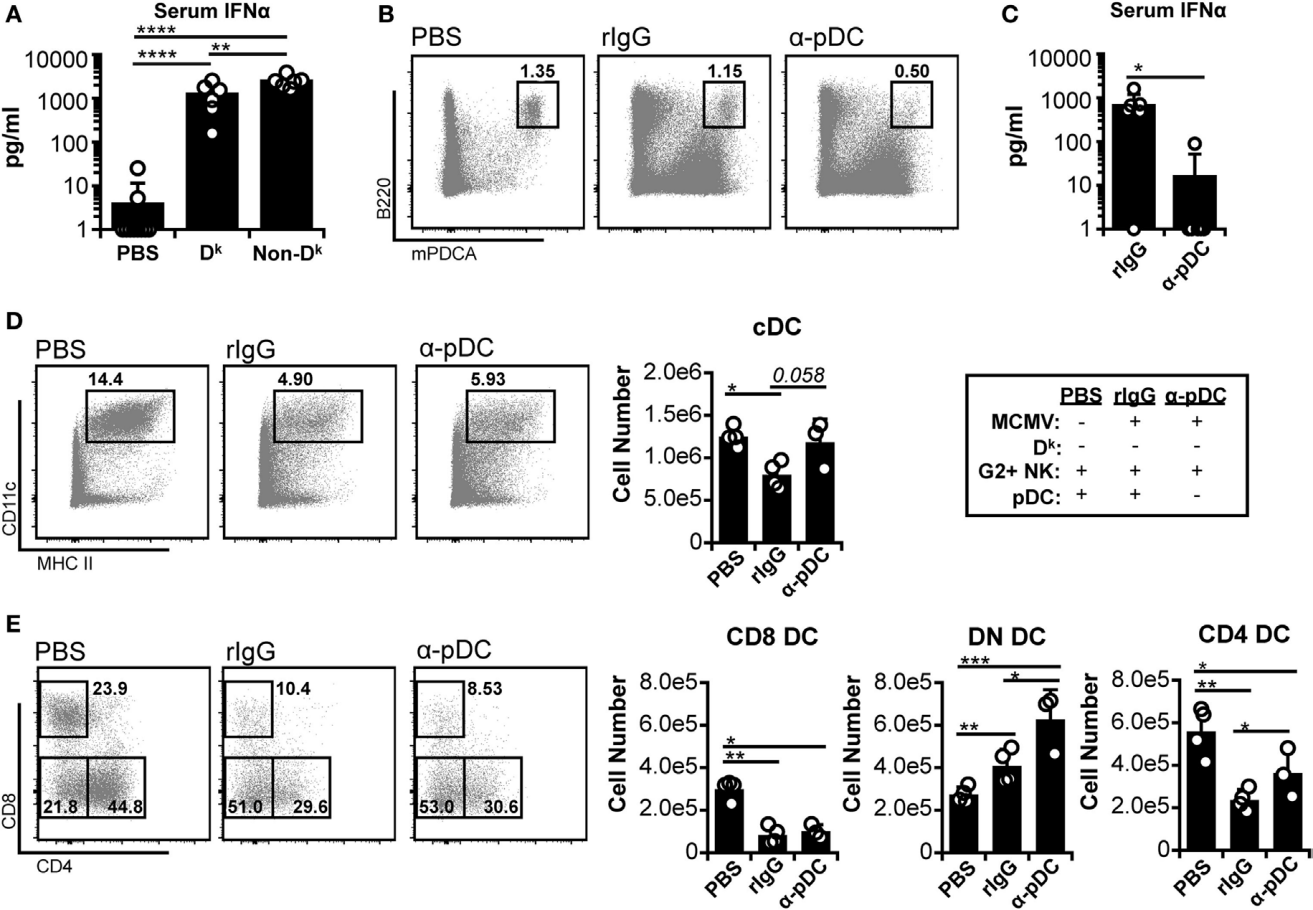
**Plasmacytoid dendritic cell (pDC) depletion reduces systemic interferon (IFN)-I and increases double-negative dendritic cell (DC) numbers, but does not restore CD8^+^ or CD4^+^ DC**. **(A)** D^k^ and non-D^k^ mice were infected with 2 × 10^4^ PFU murine cytomegalovirus (MCMV), and serum IFNα levels were assessed 36 h post infection (p.i.) by ELISA. Results are pooled from two separate experiments with three to four mice per group each. **(B–E)** Non-D^k^ mice received either isotype control (rIgG) or monoclonal antibody (mAb) 927 (α-pDC) 48 and 24 h prior to infection to deplete pDC. Mice were mock (PBS) or MCMV infected (2 × 10^4^ PFU) for 4 days. Splenocytes were stained with fluorescent mAbs and assessed for cDCs by flow cytometry. **(B)** Concatenated flow plots show pDC populations at 4 days p.i. **(C)** Graph shows serum IFNα levels at 36 h p.i. as assessed by ELISA (data pooled from two experiments). **(D)** Concatenated flow plots show gated DC populations. DC numbers are quantified in the graph. **(E)** As in **(D)** except cDC subsets are analyzed. Numbers on flow plots denote frequency of parent population. Graphs show results for individual mice (dots) and group means (bars). Results are representative of at least two experiments.

Since licensed NK cell-mediated virus control did not fully protect cDC populations, we further investigated the basis of cDC loss. It has been suggested that excessive IFN-I production from pDC could lead to cDC loss during infection (5, 10, 11). In this context, our data implied that licensed NK cells might reduce IFN-I levels enough to blunt cDC loss, but not enough to allow complete retention. Indeed, both D^k^ and non-D^k^ mice exhibited large increases in serum IFNα during early infection, but D^k^ mice produced lower amounts overall than non-D^k^ mice (Figure 2A). These data fit the hypothesis that high IFN-I could account for the residual cDC loss observed in D^k^ mice.

To further examine the role of IFN-I, we specifically depleted pDC before infection. We examined susceptible (non-D^k^) mice since the mechanism of cDC loss should be similar in D^k^ and non-D^k^ settings if IFN-I toxicity is responsible. Furthermore, cDC protection should be readily detectable in a susceptible setting since loss is profound in these mice. As expected, the α-pDC mAb treatment effectively reduced the pDC population and serum IFNα levels (Figures 2B,C). The pDC depletion also resulted in increased numbers of cDC, but the majority of the effect was due to an expanded DN DC compartment (Figures 2D,E). Neither CD8^+^ nor CD4^+^ DC numbers were fully restored by the removal of pDC. These results suggest that pDC and pDC-derived IFN-I are unlikely to be responsible for the loss of CD8^+^ and CD4^+^ DC, although they may restrict DN DC during infection.

### IFNAR Blockade Increases DN DC Numbers but Does Not Protect CD8^+^ and CD4^+^ DC

**Figure 3:**
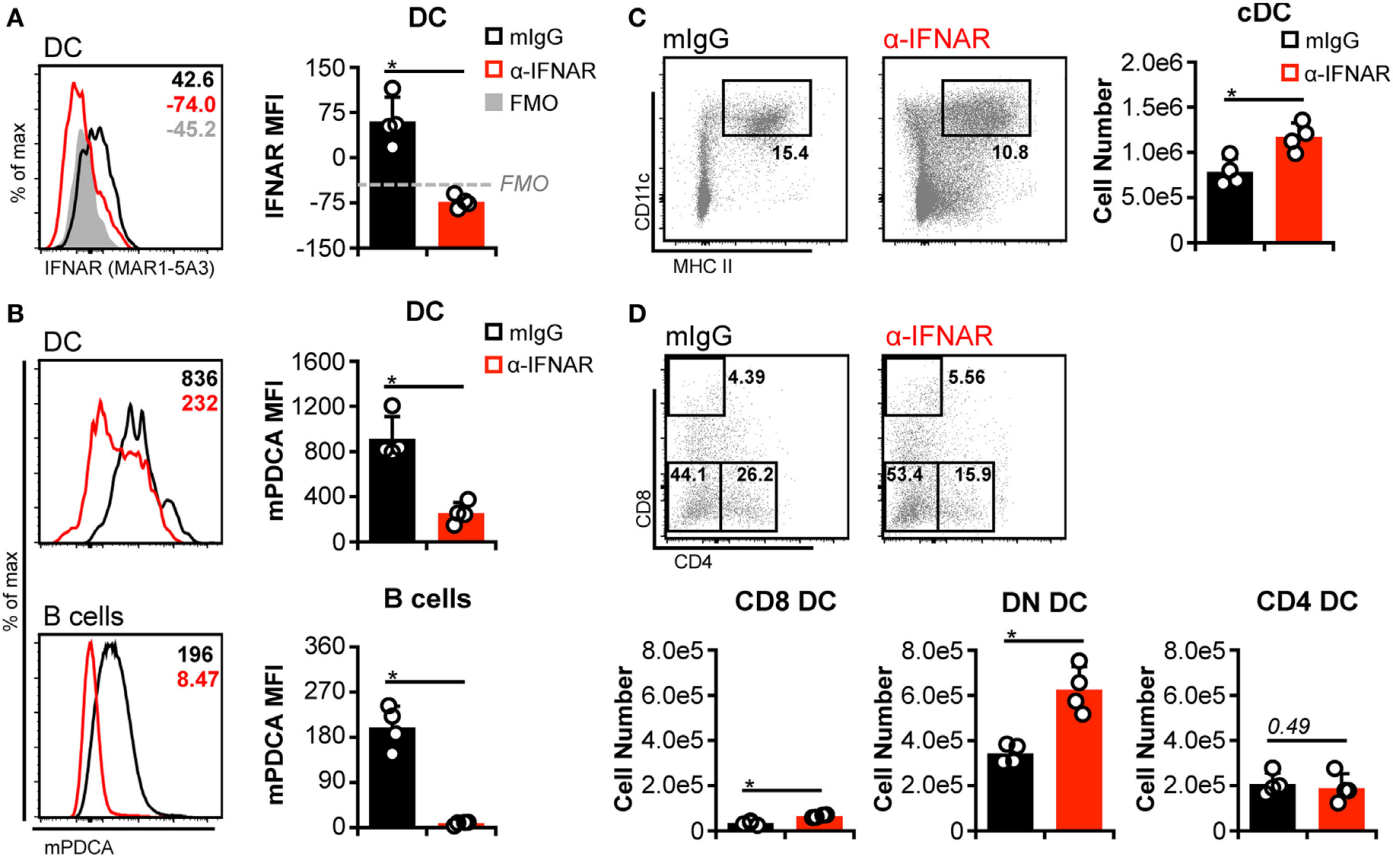
**Interferon-I receptor (IFNAR) blockade increases double-negative dendritic cells (DC) numbers but does not protect CD8^+^ and CD4^+^ DC**. Non-D^k^ mice were treated with isotype control (mIgG) or monoclonal antibody (mAb) MAR1-5A3 (α-IFNAR) prior to and throughout murine cytomegalovirus infection to block IFNAR signaling. Mice were infected for 3 days, and splenocytes were stained with fluorescent mAb and assessed by flow cytometry. **(A)** Concatenated histograms show *ex vivo* staining of DC with fluorescent α-IFNAR. Median fluorescent intensity of IFNAR staining is quantified in the graph. FMO, fluorescence minus one negative control. **(B)** As in **(A)** but data for surface mPDCA staining is shown for DC (*above*) and B cells *(below)*. **(C)** Concatenated flow plots show gated DC populations. DC numbers are quantified in the graph. **(D)** As in **(C)** except conventional DC subsets are analyzed. Numbers on flow plots denote frequency of parent population. Graphs show results for individual mice (dots) and group means (bars). Results are representative of at least two experiments.

Although pDC are responsible for the bulk of IFN-I released prior to 48 h p.i., additional cell types also produce IFN-I during the course of MCMV infection (e.g., cDC, stromal cells, macrophages) (43). Hence, these could contribute to cDC loss in the absence of pDC if cDC loss is truly dependent on IFN-I signaling. We therefore broadly impaired IFN-I responses by blocking IFNAR signaling in non-D^k^ mice. Receptor occupancy was assessed by staining DC with fluorophore-conjugated α-IFNAR. As expected, IFNAR-blocked DC were much less effectively stained than DC from isotype-treated mice (Figure 3A). Inhibition of IFNAR signaling was verified by measuring mPDCA upregulation on DC and B cells, since surface mPDCA expression is highly responsive to IFN-I stimulation (44). Cells from IFNAR-blocked mice expressed much lower levels of mPDCA in comparison to isotype-treated controls (Figure 3B). Expression of the activation markers CD69 and CD86 was also much lower on T cells, B cells, and/or DC from IFNAR-blocked mice (Figure S5 in Supplementary Material).

Similar to pDC depletion, we observed a greater number of cDC in IFNAR-blocked mice (Figure 3C). However, this again stemmed largely from an increase in DN DC (Figure 3D). Although higher CD8^+^ DC numbers were also detected in IFNAR-blocked spleens, their overall retention was negligible when compared to numbers observed in mock-infected mice (Figure 3D compared to PBS in Figures 1 and 2). CD4^+^ DC numbers were unaffected by IFNAR blockade and declined equally in all mice during MCMV infection (Figure 3D compared to PBS in Figures 1 and 2). In aggregate, these results indicated that the loss of CD8^+^ and CD4^+^ DCs during MCMV infection is largely IFN-I independent, but IFN-I signaling specifically limits DN DC numbers.

### MCMV-Induced Loss of CD8^+^ and CD4^+^ DC Persists in the Absence of IFNAR Signaling

**Figure 4:**
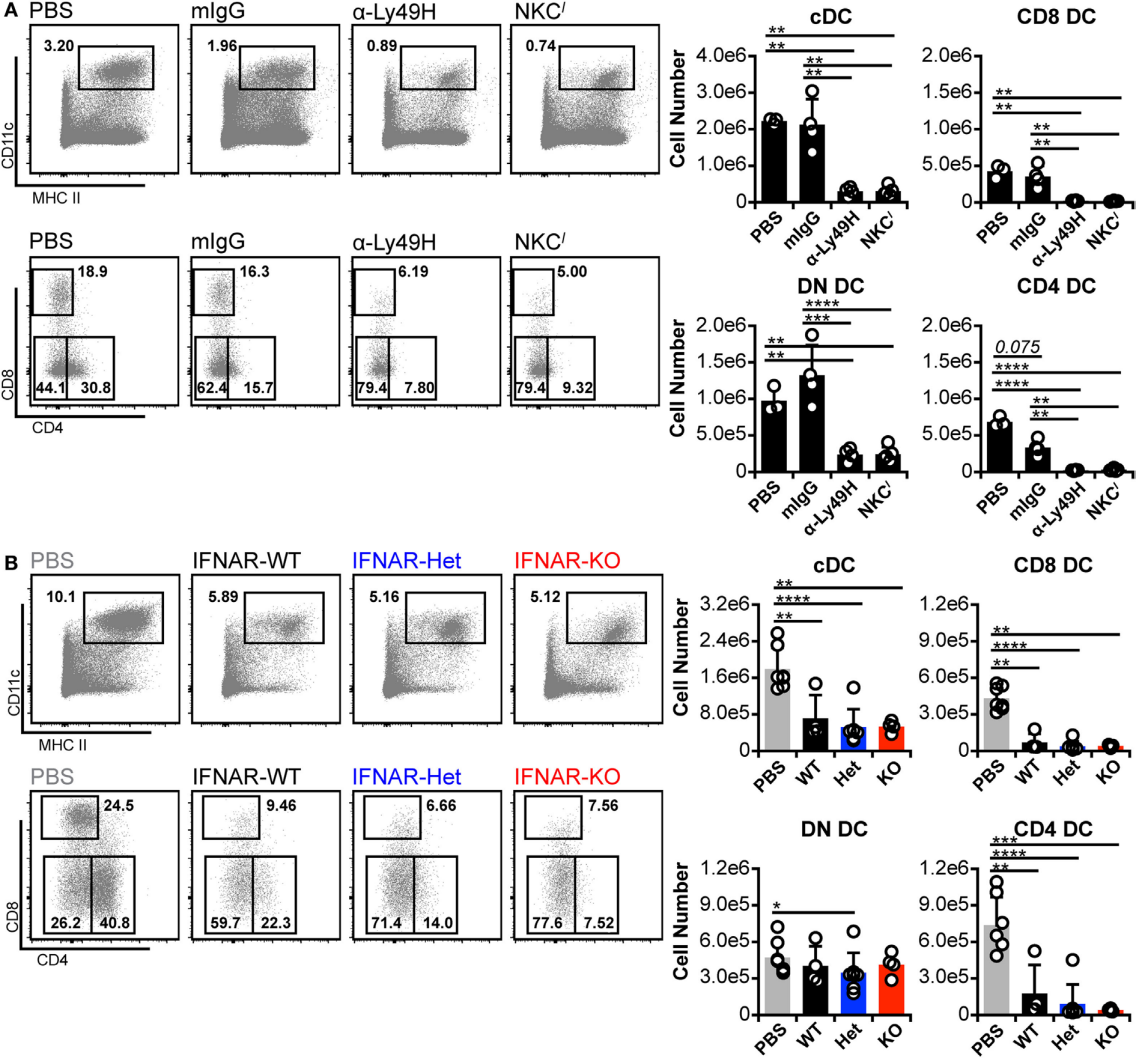
**Murine cytomegalovirus (MCMV)-induced loss of CD8^+^ and CD4^+^ dendritic cell (DC) persists in the absence of interferon-I receptor (IFNAR) signaling**. B6 and B6-derived mice were mock (PBS) or MCMV infected for 3 days. Infected mice were either B6 WT controls (mIgG), Ly49H-blocked B6 WT (α-Ly49H), or Ly49H-deficient B6 congenic mice (NKC*^l^*). Splenocytes were stained with fluorescent monoclonal antibodies (mAb) and assessed for cDCs by flow cytometry. **(A)** Concatenated flow plots show gated DCs (*above*) and cDC subsets (*below*). Cell numbers for each population are quantified in the graphs. **(B)** Ly49H-deficient mice were bred with IFNAR-KO B6 mice to generate B6.NKC*^l^* with WT (IFNAR-WT), heterozygous (IFNAR-Het), or null (IFNAR-KO) IFNAR expression. Mice were mock (PBS) or MCMV infected for 3 days, and splenocytes were stained with fluorescent mAb for analysis. Concatenated flow plots show DC (*above*) and cDC subsets (*below*) for each group. Cell numbers for each population are quantified in the graphs. Numbers on flow plots denote frequency of parent population. Graphs show results for individual mice (dots) and group means (bars). Results are representative of at least two experiments.

To exclude the possibility of low-level IFN-I signaling despite IFNAR blockade, we repeated the experiments in IFNAR-deficient (IFNAR-KO) C57BL/6 (B6) mice. Although we had thus far examined cDC in C57L-derived mice, C57L and B6 mice are genealogically related (45), but encode distinct Ly49 receptors in their respective NKC haplotypes (46–49). Notably, the B6 NKC encodes Ly49H, which mediates MCMV resistance (19–23), whereas C57L mice lack Ly49H expression. We therefore mitigated this NKC difference in our experiments by Ly49H blockade or congenic replacement of the B6 NKC with the C57L NKC (NKC*^l^* mice; see Materials and Methods). When cDC loss was assessed in the B6 background, isotype-treated, Ly49H-sufficient mice retained total cDC, CD8^+^ DC, and DN DC numbers (Figure 4A). Unexpectedly, although, they exhibited a strong trend toward loss of CD4^+^ DC, emphasizing the sensitivity of this population to infection-induced loss (Figure 4A). The reduced number of CD4^+^ DC was apparently compensated for by a slight increase in DN DC—thereby maintaining total cDC numbers. B6 mice lacking Ly49H-mediated resistance (α-Ly49H and NKC*^l^*) displayed pronounced overall loss of cDC numbers (Figure 4A). This included significant decreases in CD8^+^ and CD4^+^ DC but, unlike C57L-derived mice, also included significant loss of DN DC numbers (Figure 4A). These data showed that, when B6 mice lack Ly49H-mediated resistance, MCMV-induced cDC loss is at least similar to that observed on the C57L background and, potentially, more profound given the DN DC data.

We crossed B6.IFNAR-KO and NKC*^l^* mice to generate B6.NKC*^l^* IFNAR-KO animals, which lack both Ly49H and IFNAR expressions (IFNAR-KO). This also yielded IFNAR-WT and IFNAR-Het littermates (Figure S6 in Supplementary Material), which were used as comparators. Upon challenge with MCMV, none of the mice displayed enhanced cDC retention, regardless of IFNAR expression status (Figure 4B). This contrasts somewhat with results from C57L mice, which maintained cDC numbers following IFNAR blockade due to an increase in DN DC. The lack of cDC retention was accompanied by a corresponding failure to increase DN DC numbers (Figure 4B). It is unclear why DN DC behaved differently in these IFNAR-KO mice, but such results could indicate that these cells are programmed differently when IFNAR signaling is absent throughout development rather than transiently blocked on mature cells. Alternatively, additional genetic modifiers could have altered DN DC behavior in this setting. Nonetheless, CD8^+^ and CD4^+^ DC loss was also comparable across all IFNAR genotypes (Figure 4B). The results demonstrated that cDC loss progressed similarly in the presence or absence of IFNAR signaling.

### Cell-Intrinsic IFNAR Deficiency Does Not Promote cDC Subset Retention

**Figure 5:**
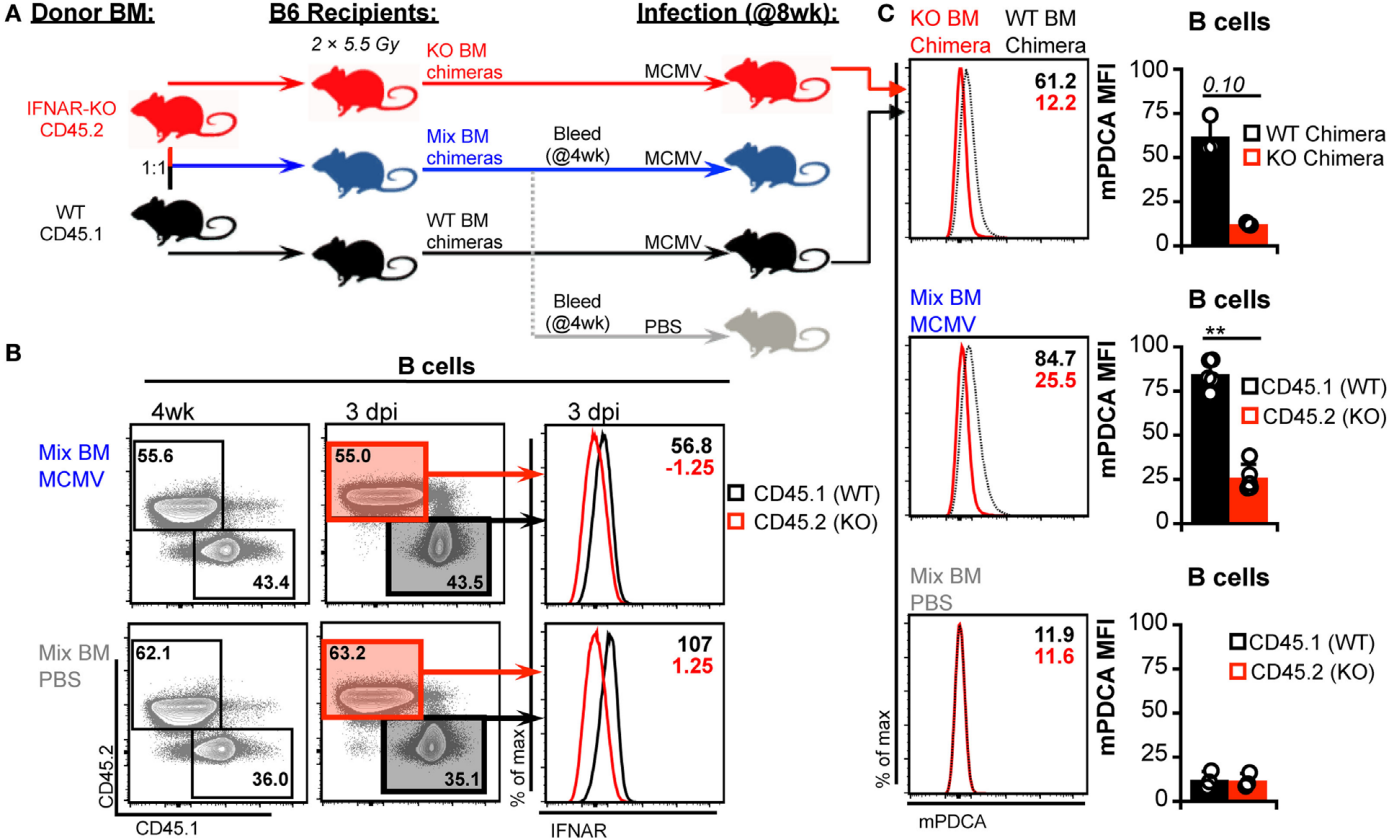
**Generation and characterization of bone marrow (BM) chimeras**. **(A)** Schematic for generation and analysis of BM chimeric mice. Irradiated B6 recipients were reconstituted with interferon-I receptor (IFNAR)-KO BM (45.2), IFNAR wild-type (IFNAR-WT) BM (45.1), or a 1:1 mix of both. Mixed chimerism was assessed in peripheral blood at 4 week postreconstitution. At 8 week postreconstitution, mice were mock (PBS) or murine cytomegalovirus (MCMV) infected for 3 days. A subgroup of the mixed chimeras were used as the PBS controls. Splenocytes were stained with fluorescent monoclonal antibodies for analysis by flow cytometry. **(B)** Concatenated flow plots show staining of CD45.2 and CD45.1 on B cells from mixed BM chimeras. Analysis was performed on peripheral blood at 4 week postreconstitution (*left*) and on spleen cells at the conclusion of the experiment (*right*). Concatenated histograms show IFNAR expression profiles of the CD45.1 versus CD45.2 marked cells (*far right*). **(C)** Surface mouse pDC antigen (mPDCA) expression was assessed on splenic B cells 3 days post infection. Concatenated histograms show surface mPDCA expression, and graphs show quantified MFI values. Top panels depict B cells from IFNAR-WT chimeras alongside B cells from IFNAR-KO chimeras. Middle panels depict CD45.1 and CD45.2 cells from infected mixed chimeras. Bottom panels depict CD45.1 and CD45.2 cells from PBS-treated mixed chimeras. All graphs show data for individual mice (dots) and group means (bars). Results are representative of at least two experiments.

Global blockade and genetic ablation of IFN-I signaling can broadly alter cytokine profiles, immune cell responses, and overall host resistance to viral infection—all of which could influence DC numbers (3, 43, 50, 51). Thus, we considered that such reduced virus control could induce additional mechanisms of DC loss and interfere with our ability to detect IFN-I-dependent toxicity. To verify the degree of IFN-I-intrinsic toxicity in cDC populations, we generated a panel of BM chimeras with varying combinations of IFNAR expression. B6 mice were used as BM transplant recipients to preserve WT IFNAR signaling in stromal cells. These cells are primary targets of MCMV and IFN-I signaling in this compartment is important for limiting initial virus spread (4, 52, 53). B6 hosts were reconstituted with IFNAR-WT BM (CD45.1^+^), IFNAR-KO BM (CD45.2^+^), or a 1:1 mix to generate hematopoietic cell compartments with full, null, or half IFNAR-signaling capability (Figure 5A). As expected, IFNAR-KO B cells from IFNAR-KO and mixed chimeras lacked IFNAR expression (Figure 5B) and failed to upregulate mPDCA in response to MCMV (Figure 5C). Thus, IFNAR-KO cells remained IFN-I insensitive in all chimeric settings.

**Figure 6:**
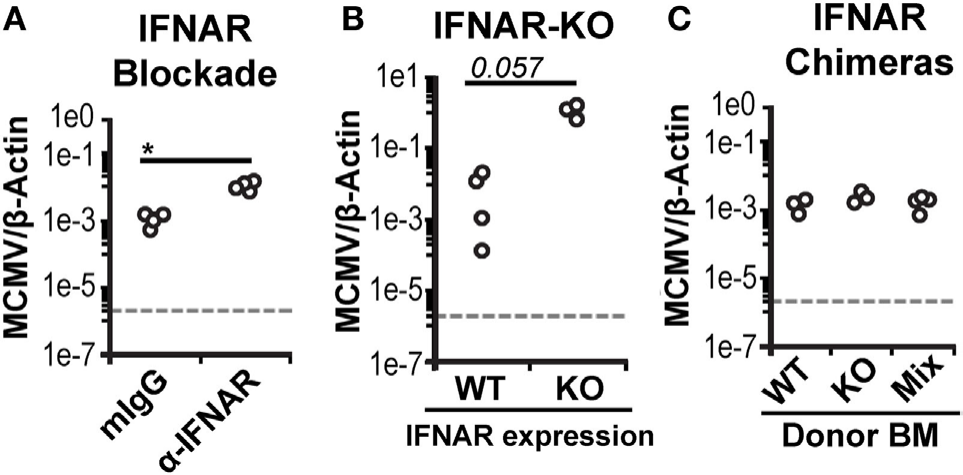
**Bone marrow (BM) chimeric mice control murine cytomegalovirus (MCMV) equally**. Viral genomes in infected spleen tissue was measured using real-time PCR and is shown as a ratio normalized by endogenous β-actin. **(A)** Virus levels in the spleens of isotype-treated (mIgG) or interferon-I receptor (IFNAR)-blocked (α-IFNAR) mice. **(B)** Virus levels in the spleens of IFNAR-WT (WT) or IFNAR-KO (KO) B6.NKC*^l^* mice. **(C)** Virus levels in the spleens of infected IFNAR-WT, IFNAR-KO, and mixed BM chimeras.

**Figure 7:**
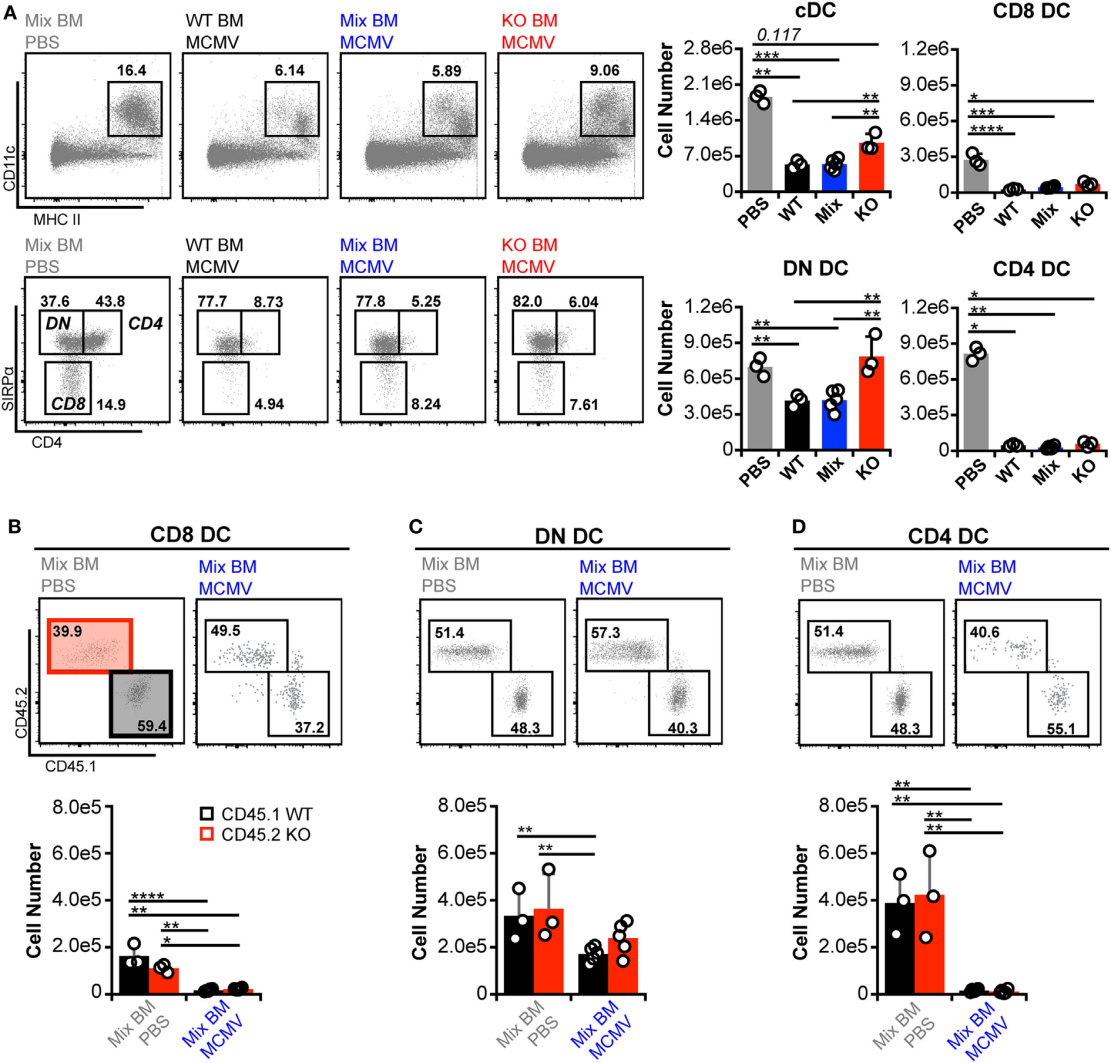
**Interferon-I receptor (IFNAR) deficiency does not provide conventional dendritic cell (cDC) subsets with an inherent retention benefit**. **(A)** Total splenocytes from mock- and murine cytomegalovirus (MCMV)-infected BM chimeras were analyzed without distinguishing between CD45.1 or CD45.2 expression. Concatenated flow plots show analysis of dendritic cell (DC) (*above*) and cDC subsets (*below*). Cell numbers for each population are quantified in the graphs. **(B–D)** cDC subsets from the mixed chimeras in **(A)** were separated into IFNAR-KO (45.2) and -WT (45.1) populations and analyzed side by side. Concatenated flow plots show the separation and relative frequency of KO versus WT cells for each subset from mock- (PBS) and MCMV-infected mixed chimeras. Cell numbers for each population are quantified in the graphs. Numbers on flow plots denote frequency of parent population. All graphs show data for individual mice (dots) and group means (bars). Results are representative of at least two experiments.

As expected, manipulation of IFN-I signaling resulted in increased virus levels (Figures 6A,B). Therefore, we also assessed MCMV genome levels in the spleens of the BM chimeras. At 3 d.p.i., the spleens of all chimeric mice exhibited similar viral burdens (Figure 6C). Hence, a difference in virus levels was not a complicating factor in the analysis of chimeric mice. We first analyzed total splenic cDC without distinguishing between CD45.1 and CD45.2 cells (bulk cDC). The cDC were substantially reduced in all infected chimeras when compared to uninfected controls (Figure 7A). However, despite the fact that cDC decreased in all infected mice, KO chimeras exhibited a significant retention over both mixed and WT settings. This, again, was solely due to retention of the DN DC subset since there was no appreciable increase in the number of CD8^+^ or CD4^+^ DC in KO chimeras (Figure 7A). Thus, hematopoietic IFNAR deficiency does not protect CD8^+^ and CD4^+^ DC, though it is sufficient to maintain DN DC numbers at levels equal to those seen in uninfected mice.

Direct comparison of CD45.1 and CD45.2 DC in mixed BM chimeras revealed that none of the subsets preferentially retained IFNAR-KO cells during infection (Figures 7B–D). However, IFNAR-KO DN DC trended toward higher numbers and did not differ significantly from either WT or KO populations in uninfected mice, which could indicate an intrinsic retention benefit. Overall, the data show that, during MCMV infection, DN DC numbers are the most responsive to IFN-I regulation, while profound CD8^+^ and CD4^+^ DC loss can occur independently of IFN-I.

## Discussion

Despite their well-established protective role during the initial stages of viral infection, IFN-I can also manifest immune regulatory or suppressive effects, depending on the target cell and signaling context (3, 54). A reduction in DC has also been observed during human inflammatory conditions (e.g., HIV infection and sepsis) and may contribute to immunosuppression during chronic infection and illness (18, 55–58). Although high IFN-I responses are a hallmark of both HIV and sepsis (59–62), a direct causal relationship between IFN-I and DC loss has not been demonstrated. Previous work with murine models has correlated high IFN-I levels with DC loss, potentially *via* direct cDC toxicity (5, 6, 11, 63, 64). This correlation seemingly indicates that blocking early IFN-I responses could preserve DC numbers. This is an important consideration from a clinical standpoint, since the ability to regulate DC numbers is relevant to a wide variety of diseases. As such, the relationship between IFN-I and DC merits better understanding. The current work demonstrates that blocking IFN-I is not sufficient to protect or restore DC numbers and, furthermore, could result in an inability to efficiently control acute infections.

Previous work with lymphocytic choriomeningitis virus (LCMV) and poly I:C has correlated IFN-I with diminished DC populations. During LCMV infection, IFNAR-KO mice show improved retention of CD4^+^, CD8^+^, and DN DC with fewer DC that become annexin V^+^ during *ex vivo* culture (11). After poly I:C treatment, IFNAR-KO mice retain CD8^+^ and CD11b^+^ DC in greater numbers than their IFNAR-WT counterparts (6). Surprisingly, we did not observe the same outcomes in IFNAR-KO mice during MCMV infection. It is unclear why our results differ from these previous studies, but inherent differences between the responses to LCMV, poly I:C, and MCMV could be responsible.

While the current work shows a decoupling of IFN-I signaling and DC loss, we did not directly assess IFN-I’s potential to specifically drive cDC loss. However, additional work using exogenous IFN-I has provided insight into this issue. Naïve mice transiently enhance annexin V and TUNEL staining on CD8^+^ splenic DC following IFN-I treatment, but simultaneously increase the turnover rate of all splenic cDC (64). Interestingly, the authors specifically note that total DC numbers never diminished in these experiments. Hence, the data are consistent with IFN-I-coordinated regulation of cDC turnover, rather than broad toxicity. On the other hand, IFN-I treatment of MCMV-infected Ly49H^+^ mice results in a decrease in both CD8^+^ and CD11b^+^ DC (5). However, the degree of enhanced DC loss is intermediate. These mice still retain greater numbers of DC than susceptible, Ly49H^−^ mice during MCMV infection. The addition of IFN-I, therefore, does not recapitulate the entirety of cDC loss triggered by inefficient MCMV control. These studies not only provide valuable insight into DC regulation by IFN-I but also highlight the importance of IFN-I-independent elements.

Although MCMV is a potent inducer of cDC loss, NK cells clearly play a role in regulating DC numbers during infection. Ly49H^+^ NK cells are well known for their efficient anti-MCMV activity, and DC numbers are preserved during Ly49H^+^ responses (5, 8, 9). Lower IFN-I production in Ly49H^+^ mice has been proposed as an explanation for this DC preservation. In C57L mice, expression of D^k^ and the C57L allele of Ly49G2 allows G2^+^ NK cells to control MCMV (12, 24, 25, 39). However, our results show that IFN-I is still substantially elevated in these resistant mice. Despite a robust IFN-I response, DC protection still occurs, which indicates that the protective effect of NK cells is more likely due to efficient virus control. Specific recognition of MCMV infection appears to be a key element of the G2^+^ NK cell effect. Thus, DC protection is not a universal function of licensed NK cells, but rather corresponds to effective NK cell-mediated virus control. The mechanism underlying G2^+^ NK-mediated MCMV control is an important area of ongoing investigation and will provide further insight into the role of licensed NK cell responses in regulating cDC populations.

The ability of G2^+^ NK cells to preserve CD8^+^ and CD4^+^ DC despite heightened IFN-I, coupled with the inability to preserve these cells by removing IFN-I signals, indicates that IFN-I toxicity is not a major driver of CD8/4 DC loss in our experiments. This opens the important issue of alternative explanations for diminished DC numbers. During MCMV infection of mixed BM chimeras, IFNAR-WT DC show increased expression in both proapoptotic and antiapoptotic genes that is not present in IFNAR-KO DC, but the frequency of apoptotic DC remains low despite an overall loss of DC numbers (65). As a result, inefficient replacement from precursors was proposed as a potential mechanism. Prolonged IFN-I exposure can interfere with the ability of BM precursors to generate DC *in vivo* (63), making this a reasonable alternative to DC toxicity and death. However, our data suggest that IFN-I effects on DC precursors are unlikely to explain DC loss. If IFN-I signals diminish or inhibit pre-DC, abrogating IFNAR signaling should restore cDC numbers. In contrast to this prediction, there was no preservation of CD8^+^ or CD4^+^ DC in our IFNAR blockade and IFNAR-KO experiments. This does not rule out potential IFN-I-independent effects on DC precursors, so inefficient development/differentiation may still play a role in DC loss. Additional explanations include cellular conversion or trafficking out of the spleen. We cannot rule out the possibility that CD8^+^ and CD4^+^ DC could alter expression of cell surface molecules, thereby taking on the appearance of another cell type, possibly even that of DN DC. Sophisticated cell tracking experiments will be needed to effectively explore these questions further.

Another important consideration is that of subset specific patterns of regulation. The current study demonstrates differential retention within CD11b^+^ DC subsets during MCMV infection. CD4^+^ DC patterns more closely resemble CD8^+^ DC, rather than their DN counterparts. DN DC numbers were generally enhanced when IFN-I signaling was impaired, but CD4^+^ DC numbers were virtually unaffected by IFN-I. In addition, DN DC exhibited less severe loss under all conditions explored in our study. This phenomenon has also been noted in mice during LCMV infection and in sepsis models (11, 58, 66). The divergence of CD4^+^ and DN DC during MCMV infection is intriguing, particularly given the functional specializations of cDC subsets. Preferential retention or loss of specific subsets during infection could influence the priming and/or differentiation of adaptive immune cells, thereby influencing the development of protective immunity.

**Figure 8:**
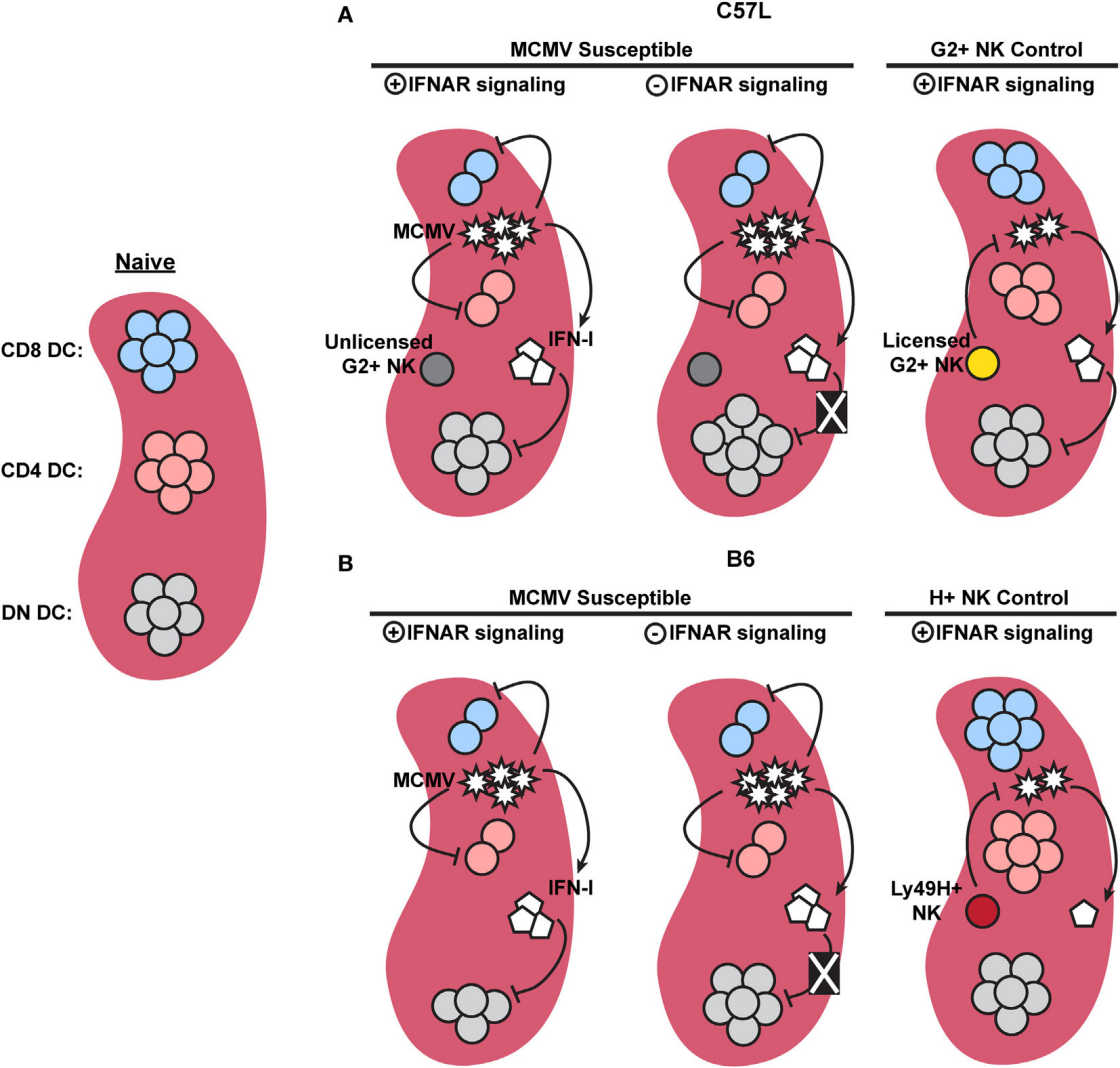
**Graphical summary showing major dendritic cell (DC) regulation in C57L and B6 mice**. **(A)** When compared to naïve mice, murine cytomegalovirus (MCMV)-infected C57L mice exhibit a decrease in CD8^+^ and CD4^+^ DC subsets, while double-negative (DN) DC numbers remain unchanged. Removal of interferon (IFN)-I signals does not restore CD8^+^ or CD4^+^ DC numbers, but does relieve an IFN-I-imposed limit on DN DC, resulting in increased numbers. Licensed Ly49G2^+^ NK-mediated MCMV control partially restores CD8^+^ and CD4^+^ DC. High levels of IFN-I, however, still specifically limits DN DC. **(B)** In the absence of Ly49H^+^ NK-mediated control, MCMV-infected B6 mice exhibit a decrease in CD8^+^, CD4^+^, and DN DC. Similar to the C57L setting, removal of IFN-I signals does not restore CD8^+^ or CD4^+^ DC, but alleviates the IFN-I-imposed restriction on DN DC. During Ly49H^+^ NK-mediated MCMV control, both virus levels and IFN-I are decreased compared to susceptible mice, resulting in preservation of all three conventional DC subsets.

Overall, the studies discussed above, in combination with the current work, emphasize that IFN-I-independent mechanisms of cDC regulation require investigation. We have clearly shown that cDC subset numbers are differentially affected by MCMV infection and IFN-I signaling (Figure 8), but that the majority of cDC loss can progress independently of IFNAR stimulation. We also showed that licensed NK cells were able to provide a degree of protection to cDC. This occurred in the presence of sustained IFN-I levels, which further shows that there are IFN-I-independent methods for manipulating cDC subset retention. It will be important to better understand these processes of cDC regulation to gain valuable insight into early events in pathogenesis and the development of immunity.

## Author Contributions

WN and MB designed the project and experiments; analyzed data and wrote the manuscript. WN performed the experiments with assistance and support from AG. All authors approved the final report.

## Conflict of Interest Statement

The authors declare that this research was conducted in the absence of any commercial or financial relationships that could be construed as a potential conflict of interest.
