# Supplementary material for: Murine Cytomegalovirus Disrupts Splenic Dendritic Cell Subsets via Type I Interferon-Dependent and -Independent Mechanisms
Source: Front Immunol. 2017 Mar 9;8:251. doi: 10.3389/fimmu.2017.00251 (PMC5343017; doi:10.3389/fimmu.2017.00251)

Supplementary Material

Murine CMV disrupts splenic dendritic cell subsets via type I interferon-dependent and independent mechanisms

William T. Nash, Alyssa L. Gillespie, Michael G. Brown*

*** Correspondence:** Corresponding Author: mgbrown@virginia.edu

**Supplementary Figure 1.** Multi-color flow cytometric gating strategies for immune cells. Fluorescent mAb stained splenocytes were run on a flow cytometer and analyzed using FlowJo v10 software. Population gates are labeled on each plot. Gating progression is shown for **(A)** total cDC, CD8 DC, CD4 DC, and DN DC; **(B)** pDC; **(C)** IFNAR on splenocytes and DC; and CD86, CD69, and mPDCA on B cells, T cells, and DC. Since inflammatory monocytes/DC (iMono) can contaminate the CD11c+ MHC II+ DC gate, these cells were excluded from numbers calculations by their profile of high side scatter and CD11b expression as shown in (A).


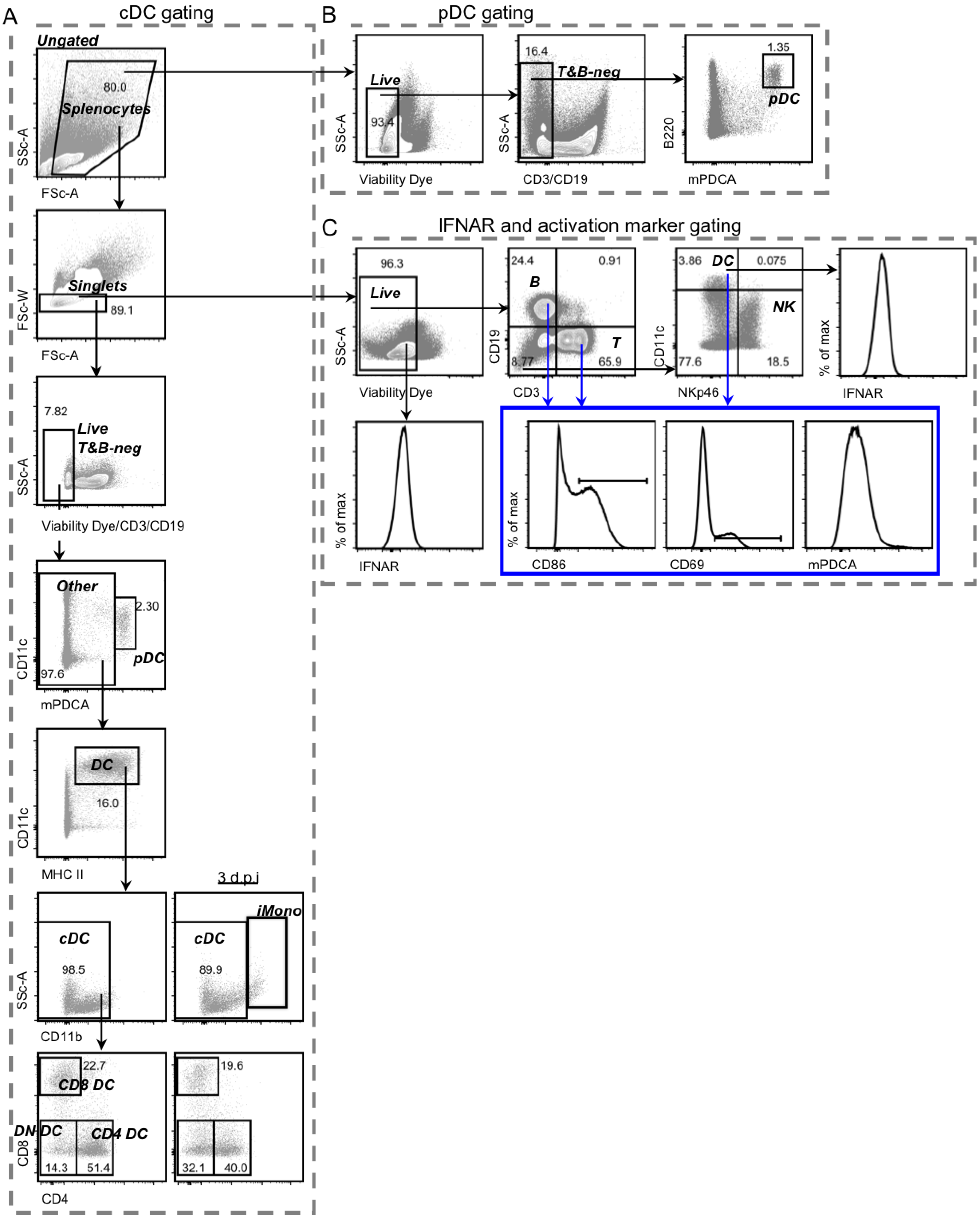


**Supplementary Figure 2.** Multi-color flow cytometric gating strategies for BM chimera experiments. Fluorescent mAb stained splenocytes were run on a flow cytometer and analyzed using FlowJo v10 software. Inclusion of mAbs to CD45.1 and CD45.2 necessitated adjustments to the panel of mAbs used for cDC detection in Fig. S1. Population gates are labeled on each plot. **(A)** Gating progression for total cDC, cDC subsets, and CD45.1/CD45.2 congenic markers. Deficiency in hematopoietic IFNAR signaling in the IFNAR-KO chimeras caused an apparent increase in the influx of inflammatory cells into the cDC gate. These cells were again excluded from analyses by their high side scatter and high CD11b profile. **(B)** Gating progression for verification of chimerism on tail blood after 4 wk of reconstitution *(left)* and splenocytes after 8 weeks of reconstitution and 3 days of MCMV infection.


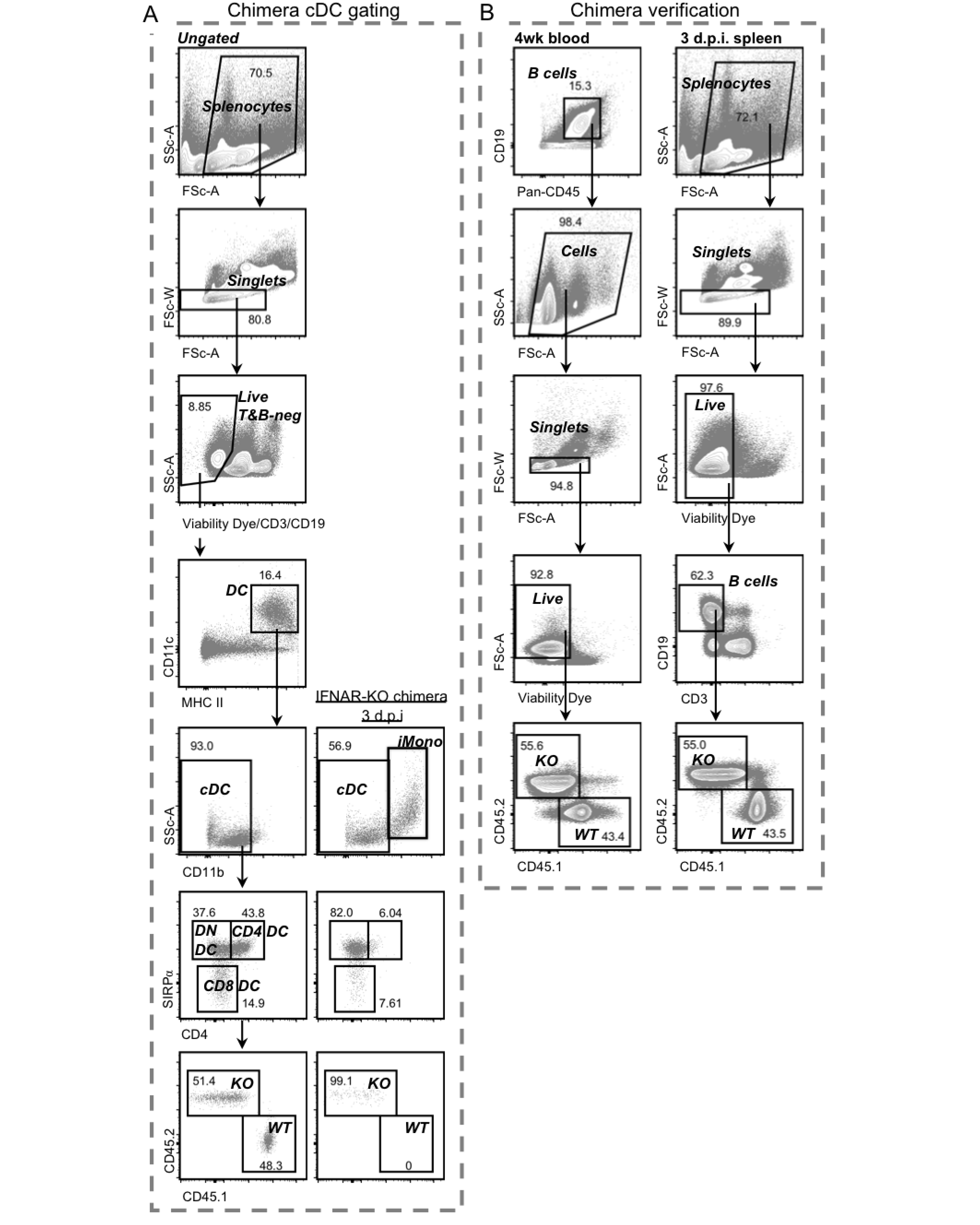


**Supplementary Figure 3.** Verification of CD8+ DC data from BM chimeras. Chimeras were generated, infected, and analyzed as in Fig. 5. At 3 days p.i., splenocytes were stained with a fluorescent mAb panel designed for the specific analysis of CD8+ DC. **(A)** Gating strategy for CD8+ DC analysis. **(B)** Concatenated flow plots show gated CD8+ DC (*left*) and separation into CD45.1/CD45.2 populations (*right*) for mock- (PBS) and MCMV-infected mixed BM chimeras. **(C)** Graph shows quantified total CD8+ DC numbers (45.1 and 45.2 combined) from all chimeric settings. **(D)** Graph shows quantified numbers of CD45.1 (IFNAR-WT) vs CD45.2 (IFNAR-KO) CD8+ DC from mock- (PBS) and MCMV-infected mixed BM chimeras. All graphs show data for individual mice (dots) and group means (bars). Numbers on flow plots denote frequency of parent population. Results are representative of at least two experiments.


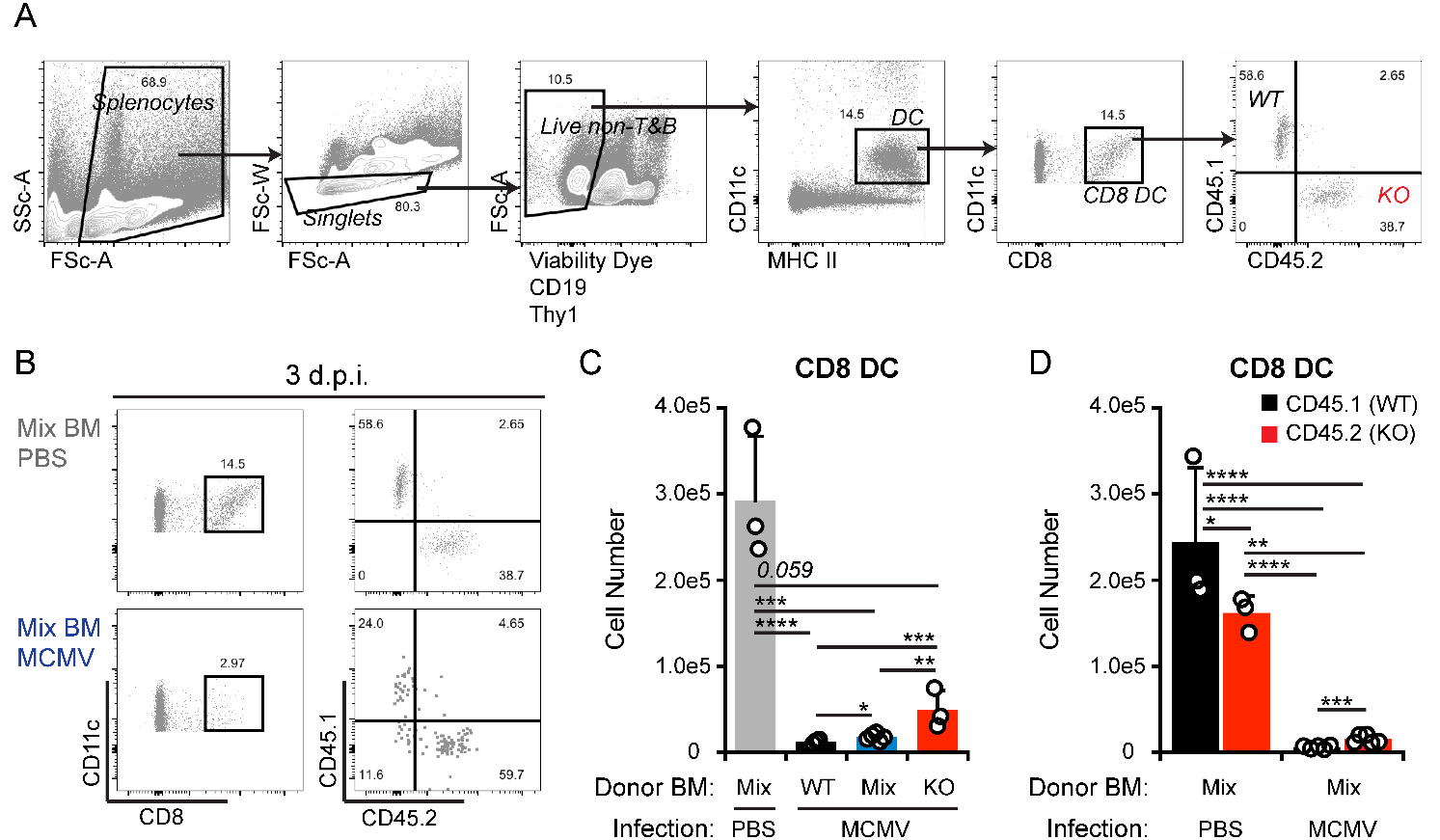


**Supplementary Figure 4.** Impact of Ly49G2 depletion on DC and NK cell subsets. **(A)** Mice were treated with the G2-depleting mAb clone 4D11 (α-G2) or isotype control (rIgG) for about 3 days and DC numbers were assessed via flow cytometry. **(B)** Mice were α-G2 or rIgG treated 2 days prior to MCMV infection. At 36 h.p.i., NKp46+ splenocytes were evaluated for Ly49 populations via flow cytometry (gating progression: live cells > singlets > NKp46+ > 12A8+/- > other Ly49 populations). Antibodies used for defining NK populations in the C57L background, which harbors a 129-like NKC, were 29A1.4 (NKp46), 12A8 (Ly49R), 14B11 (Ly49I/U), 4E5 (Ly49R/O/V), YE1/48 (Ly49P/V/R/T), and 4D11 (Ly49G/T; no MHC ligand has been identified for Ly49T and it is unclear if this inhibitory receptor is expressed at appreciable levels *in vivo.* Hence 4D11 staining and depletion phenotypes are generally attributed to Ly49G). Numbered populations on the flow plots correspond to the labels along the x-axis of the accompanying graph. All graphs show data for individual mice (dots) and group means (bars).


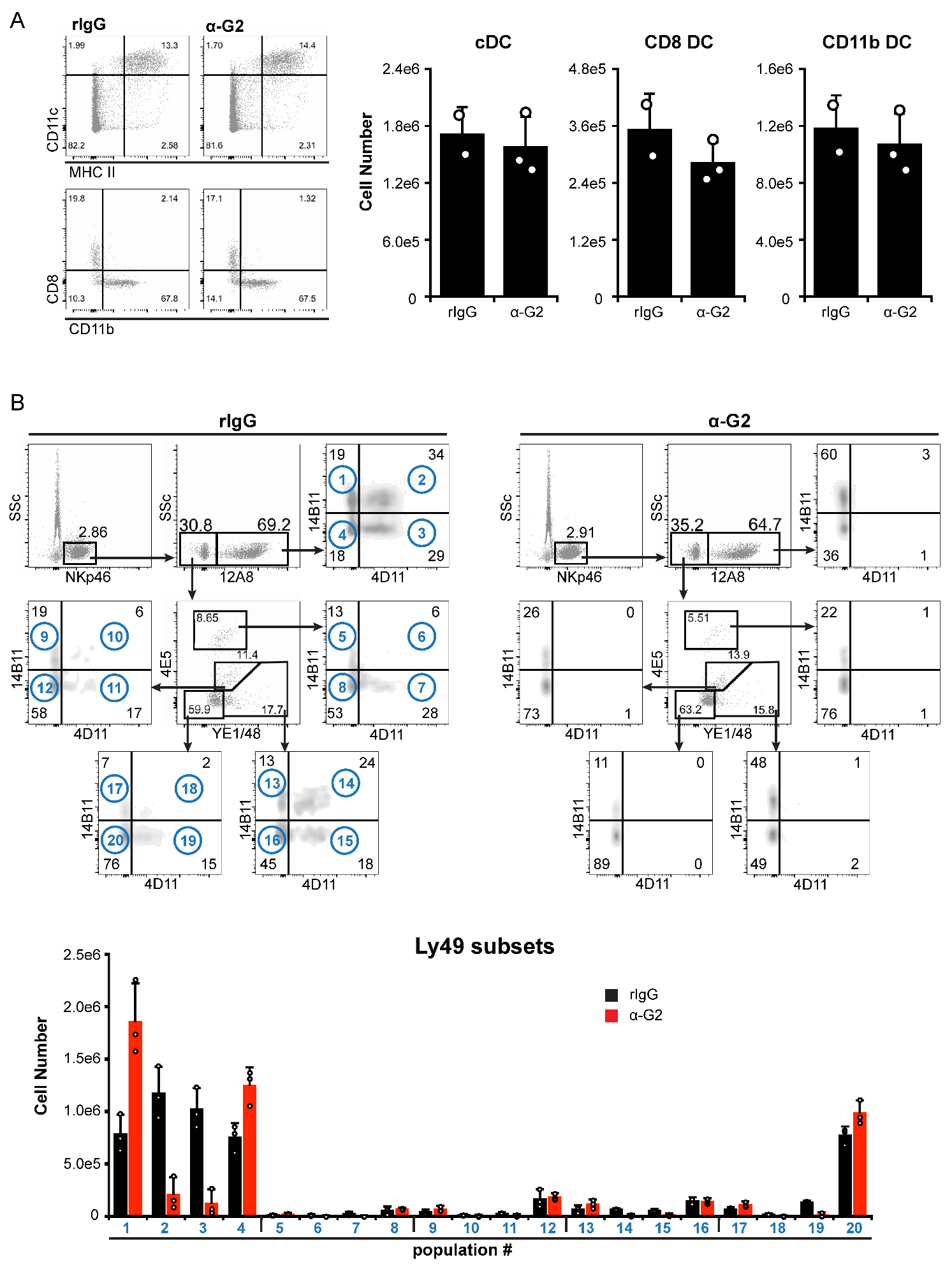


**Supplementary Figure 5.** Evaluation of activation marker expression on B cells, T cells, and DC during IFNAR blockade. Non-D^k^ mice were treated with isotype control (mIgG) or mAb MAR1-5A3 (α-IFNAR) prior to and throughout MCMV infection. At 3 days p.i., splenocytes were stained with fluorescent mAbs and assessed by flow cytometry. **(A)** Histograms show flow staining intensity for CD69 (*top*) and CD86 (*bottom*) expression on splenic T cells, B cells, and DC from mIgG or α-IFNAR treated mice. **(B)** Quantification of staining intensity from (A). All graphs show data for individual mice (dots) and group means (bars).


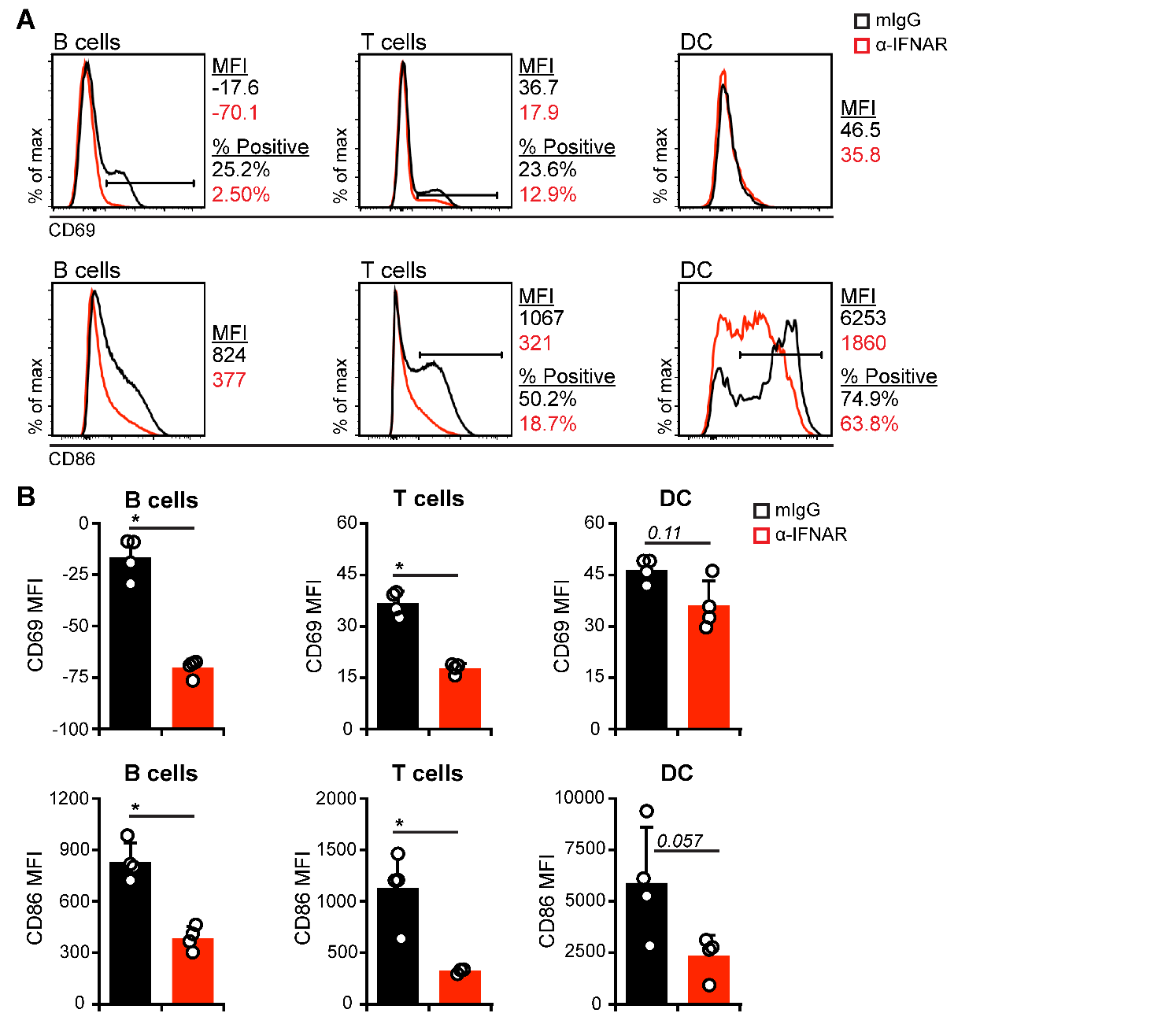


**Supplementary Figure 6.** Characterization of IFNAR-KO mice and littermates. Mice from B6.NKC*^l^* x B6.IFNAR-KO crosses with the indicated IFNAR genotypes were mock- (PBS) or MCMV-infected. At 3 days p.i., splenocytes were stained with fluorescent mAbs and cDCs were assessed by flow cytometry. **(A)** Histograms show concatenated data for IFNAR expression from each experimental group. Graph shows individual IFNAR MFI (dots) and the mean MFI for each group (bars). **(B)** Histogram shows concatenated data for mPDCA expression on B cells from each experimental group. Graph shows quantified mPDCA MFI data for individual mice (dots) as well as the mean MFI for each group (bars). Results are representative of at least two experiments.


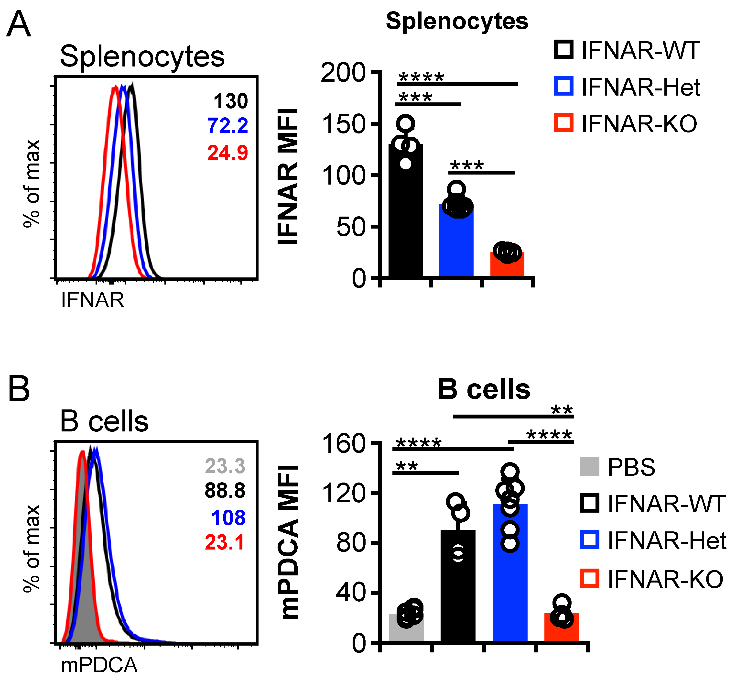

Supplement: Supplementary file 1 [file Data_Sheet_1.docx]
